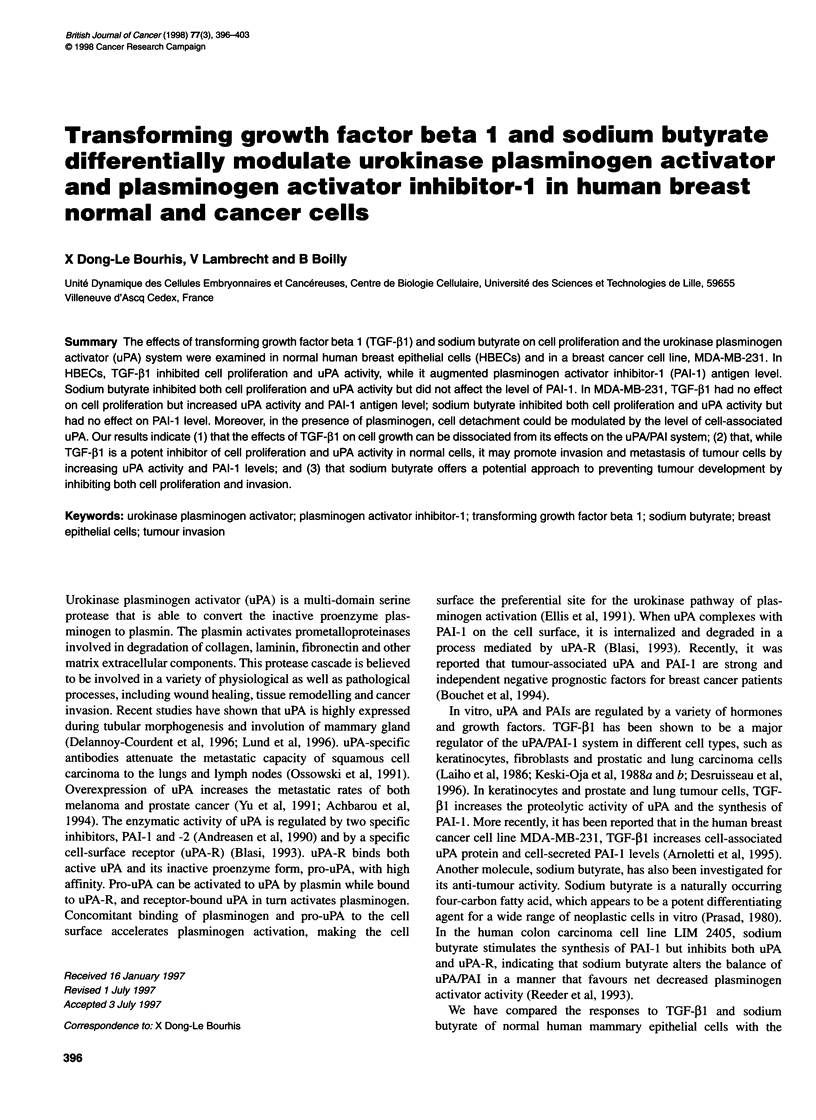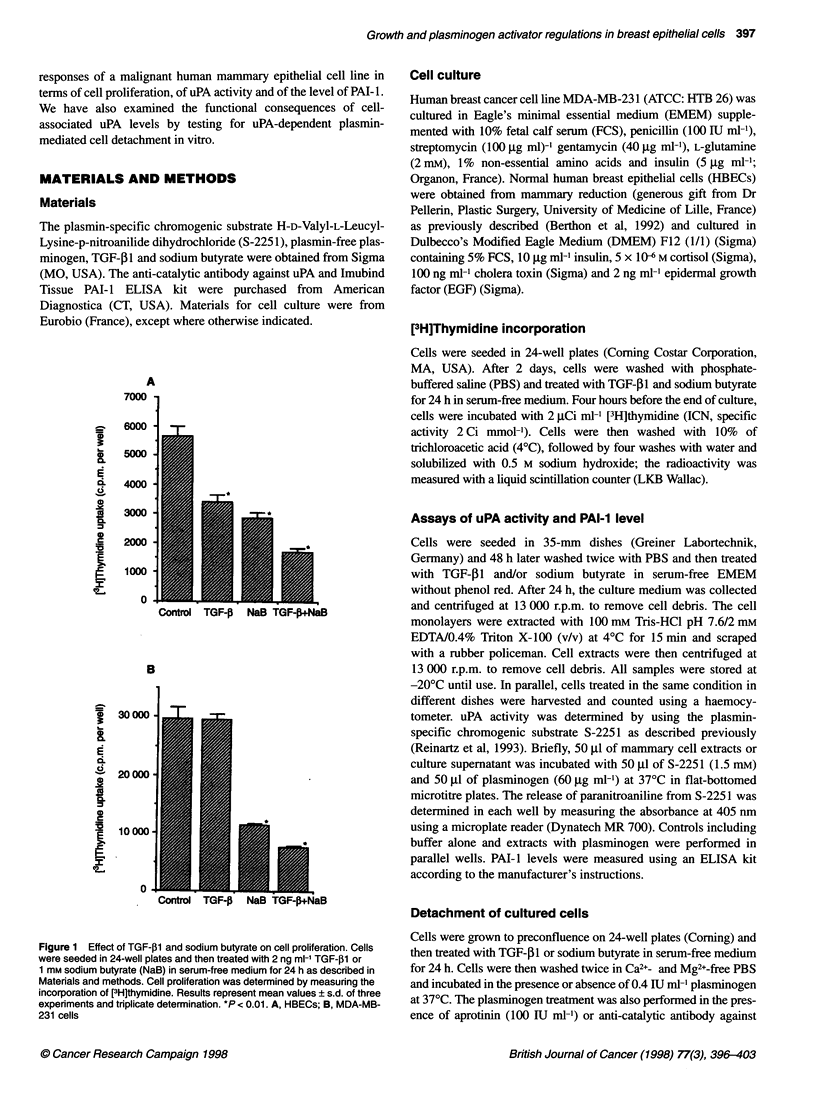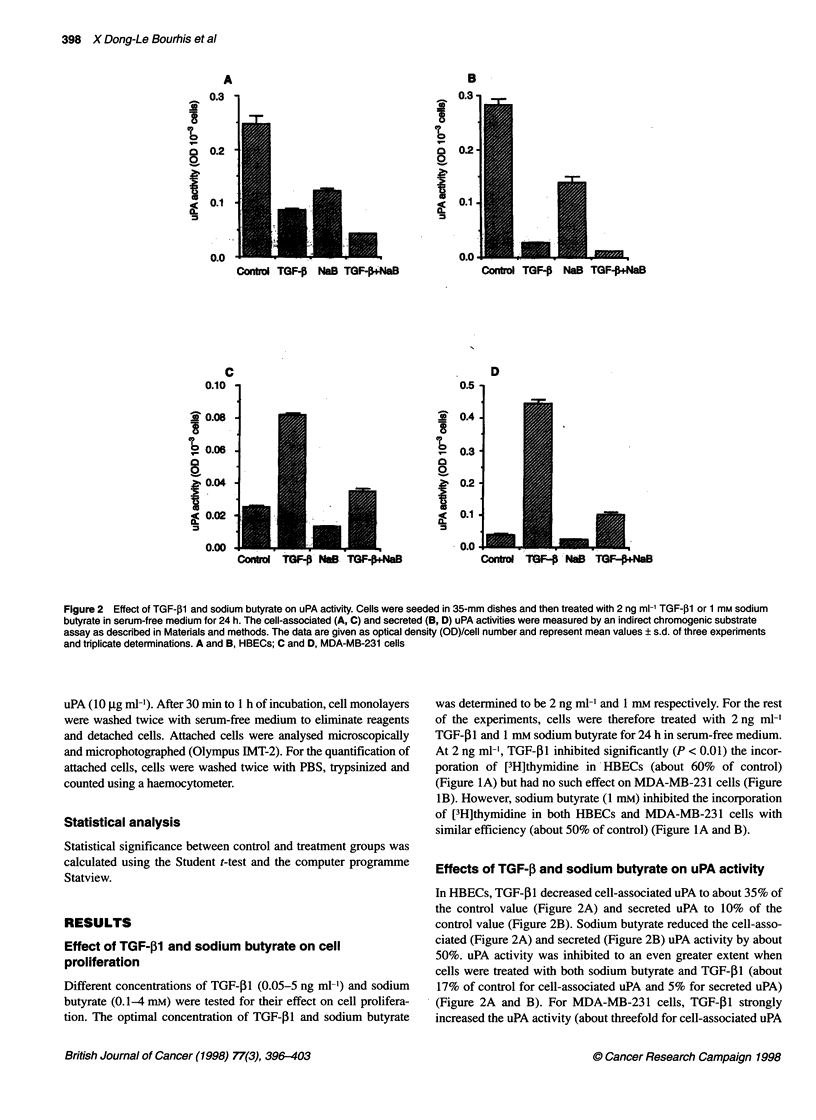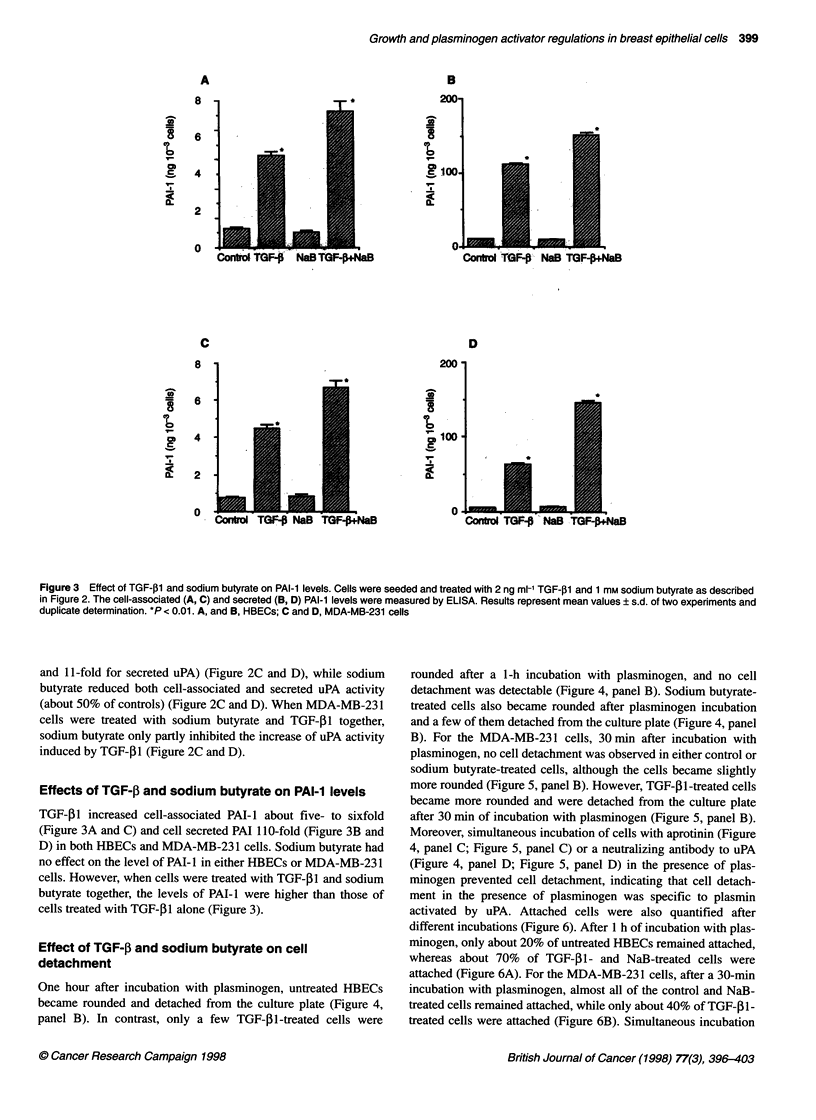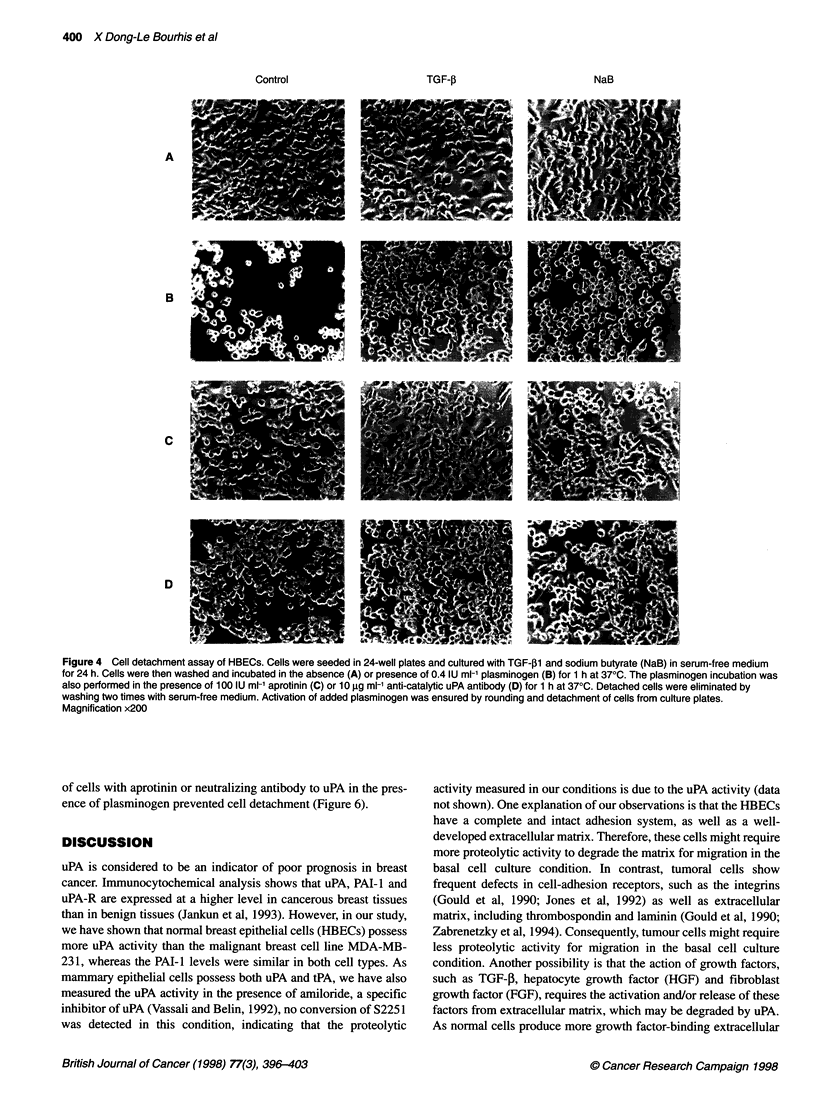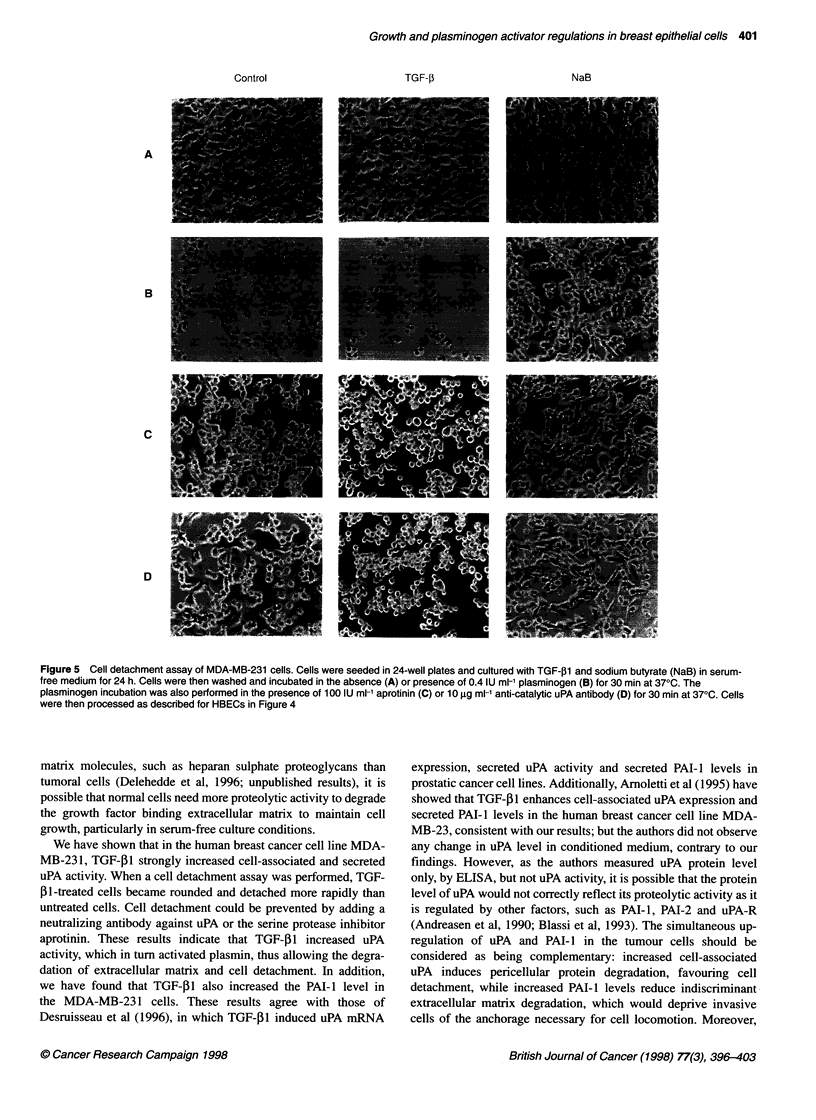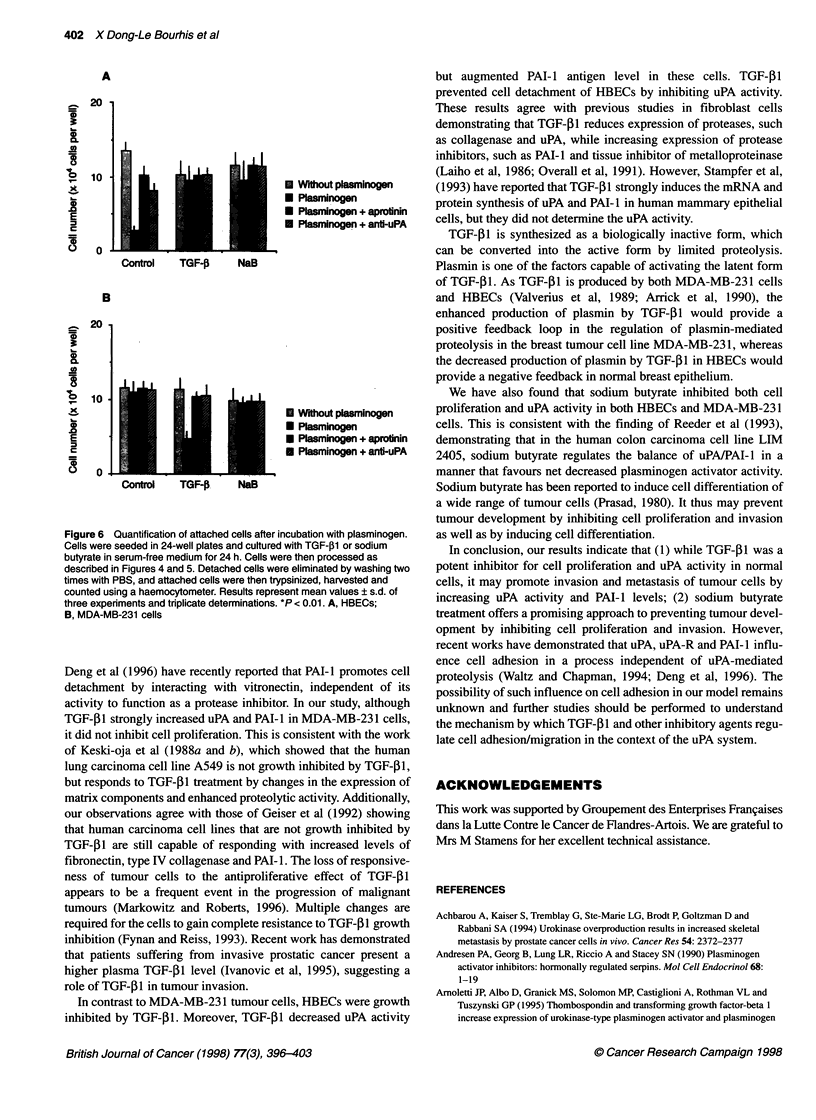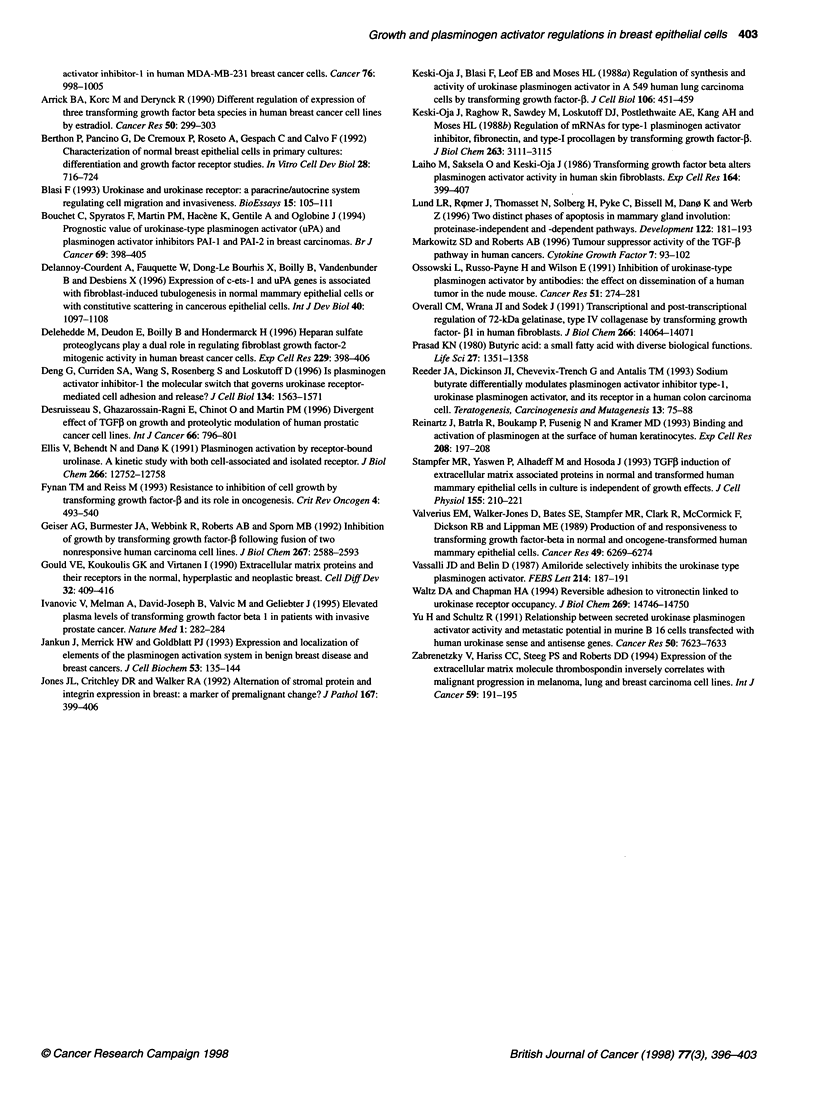# Transforming growth factor beta 1 and sodium butyrate differentially modulate urokinase plasminogen activator and plasminogen activator inhibitor-1 in human breast normal and cancer cells.

**DOI:** 10.1038/bjc.1998.63

**Published:** 1998

**Authors:** X. Dong-Le Bourhis, V. Lambrecht, B. Boilly

**Affiliations:** Centre de Biologie Cellulaire, UniversitÃ© des Sciences et Technologies de Lille, Villeneuve d'Ascq, France.

## Abstract

**Images:**


British Journal of Cancer (1998) 77(3), 396–403
© 1998 Cancer Research Campaign

### Transforming growth factor beta 1 and sodium butyrate differentially modulate urokinase plasminogen activator and plasminogen activator inhibitor-1 in human breast normal and cancer cells

**X Dong-Le Bourhis, V Lambrecht and B Boilly**

Unité Dynamique des Cellules Embryonnaires et Cancéreuses, Centre de Biologie Cellulaire, Université des Sciences et Technologies de Lille, 59655 Villeneuve d'Ascq Cedex, France

**Summary** The effects of transforming growth factor beta 1 (TGF-β1) and sodium butyrate on cell proliferation and the urokinase plasminogen activator (uPA) system were examined in normal human breast epithelial cells (HBECs) and in a breast cancer cell line, MDA-MB-231. In HBECs, TGF-β1 inhibited cell proliferation and uPA activity, while it augmented plasminogen activator inhibitor-1 (PAI-1) antigen level. Sodium butyrate inhibited both cell proliferation and uPA activity but did not affect the level of PAI-1. In MDA-MB-231, TGF-β1 had no effect on cell proliferation but increased uPA activity and PAI-1 antigen level; sodium butyrate inhibited both cell proliferation and uPA activity but had no effect on PAI-1 level. Moreover, in the presence of plasminogen, cell detachment could be modulated by the level of cell-associated uPA. Our results indicate (1) that the effects of TGF-β1 on cell growth can be dissociated from its effects on the uPA/PAI system; (2) that, while TGF-β1 is a potent inhibitor of cell proliferation and uPA activity in normal cells, it may promote invasion and metastasis of tumour cells by increasing uPA activity and PAI-1 levels; and (3) that sodium butyrate offers a potential approach to preventing tumour development by inhibiting both cell proliferation and invasion.

**Keywords:** urokinase plasminogen activator; plasminogen activator inhibitor-1; transforming growth factor beta 1; sodium butyrate; breast epithelial cells; tumour invasion

Urokinase plasminogen activator (uPA) is a multi-domain serine protease that is able to convert the inactive proenzyme plasminogen to plasmin. The plasmin activates prometalloproteinases involved in degradation of collagen, laminin, fibronectin and other matrix extracellular components. This protease cascade is believed to be involved in a variety of physiological as well as pathological processes, including wound healing, tissue remodelling and cancer invasion. Recent studies have shown that uPA is highly expressed during tubular morphogenesis and involution of mammary gland (Delannoy-Courdent et al, 1996; Lund et al, 1996). uPA-specific antibodies attenuate the metastatic capacity of squamous cell carcinoma to the lungs and lymph nodes (Ossowski et al, 1991). Overexpression of uPA increases the metastatic rates of both melanoma and prostate cancer (Yu et al, 1991; Achbarou et al, 1994). The enzymatic activity of uPA is regulated by two specific inhibitors, PAI-1 and -2 (Andreasen et al, 1990) and by a specific cell-surface receptor (uPA-R) (Blasi, 1993). uPA-R binds both active uPA and its inactive proenzyme form, pro-uPA, with high affinity. Pro-uPA can be activated to uPA by plasmin while bound to uPA-R, and receptor-bound uPA in turn activates plasminogen. Concomitant binding of plasminogen and pro-uPA to the cell surface accelerates plasminogen activation, making the cell surface the preferential site for the urokinase pathway of plasminogen activation (Ellis et al, 1991). When uPA complexes with PAI-1 on the cell surface, it is internalized and degraded in a process mediated by uPA-R (Blasi, 1993). Recently, it was reported that tumour-associated uPA and PAI-1 are strong and independent negative prognostic factors for breast cancer patients (Bouchet et al, 1994).

In vitro, uPA and PAIs are regulated by a variety of hormones and growth factors. TGF-β1 has been shown to be a major regulator of the uPA/PAI-1 system in different cell types, such as keratinocytes, fibroblasts and prostatic and lung carcinoma cells (Laiho et al, 1986; Keski-Oja et al, 1988*a* and *b*; Desruisseau et al, 1996). In keratinocytes and prostate and lung tumour cells, TGF-β1 increases the proteolytic activity of uPA and the synthesis of PAI-1. More recently, it has been reported that in the human breast cancer cell line MDA-MB-231, TGF-β1 increases cell-associated uPA protein and cell-secreted PAI-1 levels (Arnoletti et al, 1995). Another molecule, sodium butyrate, has also been investigated for its anti-tumour activity. Sodium butyrate is a naturally occurring four-carbon fatty acid, which appears to be a potent differentiating agent for a wide range of neoplastic cells in vitro (Prasad, 1980). In the human colon carcinoma cell line LIM 2405, sodium butyrate stimulates the synthesis of PAI-1 but inhibits both uPA and uPA-R, indicating that sodium butyrate alters the balance of uPA/PAI in a manner that favours net decreased plasminogen activator activity (Reeder et al, 1993).

We have compared the responses to TGF-β1 and sodium butyrate of normal human mammary epithelial cells with the

*Received 16 January 1997*
*Revised 1 July 1997*
*Accepted 3 July 1997*

*Correspondence to:* X Dong-Le Bourhis responses of a malignant human mammary epithelial cell line in terms of cell proliferation, of uPA activity and of the level of PAI-1. We have also examined the functional consequences of cell-associated uPA levels by testing for uPA-dependent plasmin-mediated cell detachment in vitro.

### MATERIALS AND METHODS

### Materials

The plasmin-specific chromogenic substrate H-D-Valyl-L-Leucyl-Lysine-p-nitroanilide dihydrochloride (S-2251), plasmin-free plasminogen, TGF-β1 and sodium butyrate were obtained from Sigma (MO, USA). The anti-catalytic antibody against uPA and Imubind Tissue PAI-1 ELISA kit were purchased from American Diagnostica (CT, USA). Materials for cell culture were from Eurobio (France), except where otherwise indicated.

**Figure 1** Effect of TGF-β1 and sodium butyrate on cell proliferation. Cells were seeded in 24-well plates and then treated with 2 ng ml$^{-1}$ TGF-β1 or 1 mM sodium butyrate (NaB) in serum-free medium for 24 h as described in Materials and methods. Cell proliferation was determined by measuring the incorporation of [$^3$H]thymidine. Results represent mean values ± s.d. of three experiments and triplicate determination. **P* < 0.01. **A**, HBECs; **B**, MDA-MB-231 cells

### Cell culture

Human breast cancer cell line MDA-MB-231 (ATCC: HTB 26) was cultured in Eagle's minimal essential medium (EMEM) supplemented with 10% fetal calf serum (FCS), penicillin (100 IU ml$^{-1}$), streptomycin (100 μg ml)$^{-1}$ gentamycin (40 μg ml$^{-1}$), L-glutamine (2 mM), 1% non-essential amino acids and insulin (5 μg ml$^{-1}$; Organon, France). Normal human breast epithelial cells (HBECs) were obtained from mammary reduction (generous gift from Dr Pellerin, Plastic Surgery, University of Medicine of Lille, France) as previously described (Berthon et al, 1992) and cultured in Dulbecco's Modified Eagle Medium (DMEM) F12 (1/1) (Sigma) containing 5% FCS, 10 μg ml$^{-1}$ insulin, $5 \times 10^{-6}$ M cortisol (Sigma), 100 ng ml$^{-1}$ cholera toxin (Sigma) and 2 ng ml$^{-1}$ epidermal growth factor (EGF) (Sigma).

### [$^3$H]Thymidine incorporation

Cells were seeded in 24-well plates (Corning Costar Corporation, MA, USA). After 2 days, cells were washed with phosphate-buffered saline (PBS) and treated with TGF-β1 and sodium butyrate for 24 h in serum-free medium. Four hours before the end of culture, cells were incubated with 2 μCi ml$^{-1}$ [$^3$H]thymidine (ICN, specific activity 2 Ci mmol$^{-1}$). Cells were then washed with 10% of trichloroacetic acid (4°C), followed by four washes with water and solubilized with 0.5 M sodium hydroxide; the radioactivity was measured with a liquid scintillation counter (LKB Wallac).

### Assays of uPA activity and PAI-1 level

Cells were seeded in 35-mm dishes (Greiner Labortechnik, Germany) and 48 h later washed twice with PBS and then treated with TGF-β1 and/or sodium butyrate in serum-free EMEM without phenol red. After 24 h, the culture medium was collected and centrifuged at 13 000 r.p.m. to remove cell debris. The cell monolayers were extracted with 100 mM Tris-HCl pH 7.6/2 mM EDTA/0.4% Triton X-100 (v/v) at 4°C for 15 min and scraped with a rubber policeman. Cell extracts were then centrifuged at 13 000 r.p.m. to remove cell debris. All samples were stored at –20°C until use. In parallel, cells treated in the same condition in different dishes were harvested and counted using a haemocytometer. uPA activity was determined by using the plasmin-specific chromogenic substrate S-2251 as described previously (Reinartz et al, 1993). Briefly, 50 μl of mammary cell extracts or culture supernatant was incubated with 50 μl of S-2251 (1.5 mM) and 50 μl of plasminogen (60 μg ml$^{-1}$) at 37°C in flat-bottomed microtitre plates. The release of paranitroaniline from S-2251 was determined in each well by measuring the absorbance at 405 nm using a microplate reader (Dynatech MR 700). Controls including buffer alone and extracts with plasminogen were performed in parallel wells. PAI-1 levels were measured using an ELISA kit according to the manufacturer's instructions.

### Detachment of cultured cells

Cells were grown to preconfluence on 24-well plates (Corning) and then treated with TGF-β1 or sodium butyrate in serum-free medium for 24 h. Cells were then washed twice in $Ca^{2+}$- and $Mg^{2+}$-free PBS and incubated in the presence or absence of 0.4 IU ml$^{-1}$ plasminogen at 37°C. The plasminogen treatment was also performed in the presence of aprotinin (100 IU ml$^{-1}$) or anti-catalytic antibody against

*© Cancer Research Campaign 1998*

**Figure 2** Effect of TGF-β1 and sodium butyrate on uPA activity. Cells were seeded in 35-mm dishes and then treated with 2 ng $ml^{-1}$ TGF-β1 or 1 mM sodium butyrate in serum-free medium for 24 h. The cell-associated (**A, C**) and secreted (**B, D**) uPA activities were measured by an indirect chromogenic substrate assay as described in Materials and methods. The data are given as optical density (OD)/cell number and represent mean values ± s.d. of three experiments and triplicate determinations. **A** and **B**, HBECs; **C** and **D**, MDA-MB-231 cells uPA (10 μg $ml^{-1}$). After 30 min to 1 h of incubation, cell monolayers were washed twice with serum-free medium to eliminate reagents and detached cells. Attached cells were analysed microscopically and microphotographed (Olympus IMT-2). For the quantification of attached cells, cells were washed twice with PBS, trypsinized and counted using a haemocytometer.

### Statistical analysis

Statistical significance between control and treatment groups was calculated using the Student *t*-test and the computer programme Statview.

### RESULTS

### Effect of TGF-β1 and sodium butyrate on cell proliferation

Different concentrations of TGF-β1 (0.05–5 ng $ml^{-1}$) and sodium butyrate (0.1–4 mM) were tested for their effect on cell proliferation. The optimal concentration of TGF-β1 and sodium butyrate was determined to be 2 ng $ml^{-1}$ and 1 mM respectively. For the rest of the experiments, cells were therefore treated with 2 ng $ml^{-1}$ TGF-β1 and 1 mM sodium butyrate for 24 h in serum-free medium. At 2 ng $ml^{-1}$, TGF-β1 inhibited significantly ($P < 0.01$) the incorporation of [$^3$H]thymidine in HBECs (about 60% of control) (Figure 1A) but had no such effect on MDA-MB-231 cells (Figure 1B). However, sodium butyrate (1 mM) inhibited the incorporation of [$^3$H]thymidine in both HBECs and MDA-MB-231 cells with similar efficiency (about 50% of control) (Figure 1A and B).

### Effects of TGF-β and sodium butyrate on uPA activity

In HBECs, TGF-β1 decreased cell-associated uPA to about 35% of the control value (Figure 2A) and secreted uPA to 10% of the control value (Figure 2B). Sodium butyrate reduced the cell-associated (Figure 2A) and secreted (Figure 2B) uPA activity by about 50%. uPA activity was inhibited to an even greater extent when cells were treated with both sodium butyrate and TGF-β1 (about 17% of control for cell-associated uPA and 5% for secreted uPA) (Figure 2A and B). For MDA-MB-231 cells, TGF-β1 strongly increased the uPA activity (about threefold for cell-associated uPA

© Cancer Research Campaign 1998

**Figure 3** Effect of TGF-β1 and sodium butyrate on PAI-1 levels. Cells were seeded and treated with 2 ng ml$^{-1}$ TGF-β1 and 1 mM sodium butyrate as described in Figure 2. The cell-associated (**A, C**) and secreted (**B, D**) PAI-1 levels were measured by ELISA. Results represent mean values ± s.d. of two experiments and duplicate determination. *$P < 0.01$. **A**, and **B**, HBECs; **C** and **D**, MDA-MB-231 cells and 11-fold for secreted uPA) (Figure 2C and D), while sodium butyrate reduced both cell-associated and secreted uPA activity (about 50% of controls) (Figure 2C and D). When MDA-MB-231 cells were treated with sodium butyrate and TGF-β1 together, sodium butyrate only partly inhibited the increase of uPA activity induced by TGF-β1 (Figure 2C and D).

### Effects of TGF-β and sodium butyrate on PAI-1 levels

TGF-β1 increased cell-associated PAI-1 about five- to sixfold (Figure 3A and C) and cell secreted PAI 110-fold (Figure 3B and D) in both HBECs and MDA-MB-231 cells. Sodium butyrate had no effect on the level of PAI-1 in either HBECs or MDA-MB-231 cells. However, when cells were treated with TGF-β1 and sodium butyrate together, the levels of PAI-1 were higher than those of cells treated with TGF-β1 alone (Figure 3).

### Effect of TGF-β and sodium butyrate on cell detachment

One hour after incubation with plasminogen, untreated HBECs became rounded and detached from the culture plate (Figure 4, panel B). In contrast, only a few TGF-β1-treated cells were rounded after a 1-h incubation with plasminogen, and no cell detachment was detectable (Figure 4, panel B). Sodium butyrate-treated cells also became rounded after plasminogen incubation and a few of them detached from the culture plate (Figure 4, panel B). For the MDA-MB-231 cells, 30 min after incubation with plasminogen, no cell detachment was observed in either control or sodium butyrate-treated cells, although the cells became slightly more rounded (Figure 5, panel B). However, TGF-β1-treated cells became more rounded and were detached from the culture plate after 30 min of incubation with plasminogen (Figure 5, panel B). Moreover, simultaneous incubation of cells with aprotinin (Figure 4, panel C; Figure 5, panel C) or a neutralizing antibody to uPA (Figure 4, panel D; Figure 5, panel D) in the presence of plasminogen prevented cell detachment, indicating that cell detachment in the presence of plasminogen was specific to plasmin activated by uPA. Attached cells were also quantified after different incubations (Figure 6). After 1 h of incubation with plasminogen, only about 20% of untreated HBECs remained attached, whereas about 70% of TGF-β1- and NaB-treated cells were attached (Figure 6A). For the MDA-MB-231 cells, after a 30-min incubation with plasminogen, almost all of the control and NaB-treated cells remained attached, while only about 40% of TGF-β1-treated cells were attached (Figure 6B). Simultaneous incubation

© Cancer Research Campaign 1998

**Figure 4** Cell detachment assay of HBECs. Cells were seeded in 24-well plates and cultured with TGF-β1 and sodium butyrate (NaB) in serum-free medium for 24 h. Cells were then washed and incubated in the absence (**A**) or presence of 0.4 IU $ml^{-1}$ plasminogen (**B**) for 1 h at 37°C. The plasminogen incubation was also performed in the presence of 100 IU $ml^{-1}$ aprotinin (**C**) or 10 μg $ml^{-1}$ anti-catalytic uPA antibody (**D**) for 1 h at 37°C. Detached cells were eliminated by washing two times with serum-free medium. Activation of added plasminogen was ensured by rounding and detachment of cells from culture plates. Magnification ×200

of cells with aprotinin or neutralizing antibody to uPA in the presence of plasminogen prevented cell detachment (Figure 6).

### DISCUSSION

uPA is considered to be an indicator of poor prognosis in breast cancer. Immunocytochemical analysis shows that uPA, PAI-1 and uPA-R are expressed at a higher level in cancerous breast tissues than in benign tissues (Jankun et al, 1993). However, in our study, we have shown that normal breast epithelial cells (HBECs) possess more uPA activity than the malignant breast cell line MDA-MB-231, whereas the PAI-1 levels were similar in both cell types. As mammary epithelial cells possess both uPA and tPA, we have also measured the uPA activity in the presence of amiloride, a specific inhibitor of uPA (Vassali and Belin, 1992), no conversion of S2251 was detected in this condition, indicating that the proteolytic activity measured in our conditions is due to the uPA activity (data not shown). One explanation of our observations is that the HBECs have a complete and intact adhesion system, as well as a well-developed extracellular matrix. Therefore, these cells might require more proteolytic activity to degrade the matrix for migration in the basal cell culture condition. In contrast, tumoral cells show frequent defects in cell-adhesion receptors, such as the integrins (Gould et al, 1990; Jones et al, 1992) as well as extracellular matrix, including thrombospondin and laminin (Gould et al, 1990; Zabrenetzky et al, 1994). Consequently, tumour cells might require less proteolytic activity for migration in the basal cell culture condition. Another possibility is that the action of growth factors, such as TGF-β, hepatocyte growth factor (HGF) and fibroblast growth factor (FGF), requires the activation and/or release of these factors from extracellular matrix, which may be degraded by uPA. As normal cells produce more growth factor-binding extracellular

 © Cancer Research Campaign 1998

**Figure 5** Cell detachment assay of MDA-MB-231 cells. Cells were seeded in 24-well plates and cultured with TGF-β1 and sodium butyrate (NaB) in serum-free medium for 24 h. Cells were then washed and incubated in the absence (**A**) or presence of 0.4 IU $ml^{-1}$ plasminogen (**B**) for 30 min at 37°C. The plasminogen incubation was also performed in the presence of 100 IU $ml^{-1}$ aprotinin (**C**) or 10 μg $ml^{-1}$ anti-catalytic uPA antibody (**D**) for 30 min at 37°C. Cells were then processed as described for HBECs in Figure 4

matrix molecules, such as heparan sulphate proteoglycans than tumoral cells (Delehedde et al, 1996; unpublished results), it is possible that normal cells need more proteolytic activity to degrade the growth factor binding extracellular matrix to maintain cell growth, particularly in serum-free culture conditions.

We have shown that in the human breast cancer cell line MDA-MB-231, TGF-β1 strongly increased cell-associated and secreted uPA activity. When a cell detachment assay was performed, TGF-β1-treated cells became rounded and detached more rapidly than untreated cells. Cell detachment could be prevented by adding a neutralizing antibody against uPA or the serine protease inhibitor aprotinin. These results indicate that TGF-β1 increased uPA activity, which in turn activated plasmin, thus allowing the degradation of extracellular matrix and cell detachment. In addition, we have found that TGF-β1 also increased the PAI-1 level in the MDA-MB-231 cells. These results agree with those of Desruisseau et al (1996), in which TGF-β1 induced uPA mRNA expression, secreted uPA activity and secreted PAI-1 levels in prostatic cancer cell lines. Additionally, Arnoletti et al (1995) have showed that TGF-β1 enhances cell-associated uPA expression and secreted PAI-1 levels in the human breast cancer cell line MDA-MB-23, consistent with our results; but the authors did not observe any change in uPA level in conditioned medium, contrary to our findings. However, as the authors measured uPA protein level only, by ELISA, but not uPA activity, it is possible that the protein level of uPA would not correctly reflect its proteolytic activity as it is regulated by other factors, such as PAI-1, PAI-2 and uPA-R (Andreasen et al, 1990; Blassi et al, 1993). The simultaneous up-regulation of uPA and PAI-1 in the tumour cells should be considered as being complementary: increased cell-associated uPA induces pericellular protein degradation, favouring cell detachment, while increased PAI-1 levels reduce indiscriminant extracellular matrix degradation, which would deprive invasive cells of the anchorage necessary for cell locomotion. Moreover,

© Cancer Research Campaign 1998

**Figure 6** Quantification of attached cells after incubation with plasminogen. Cells were seeded in 24-well plates and cultured with TGF-β1 or sodium butyrate in serum-free medium for 24 h. Cells were then processed as described in Figures 4 and 5. Detached cells were eliminated by washing two times with PBS, and attached cells were then trypsinized, harvested and counted using a haemocytometer. Results represent mean values ± s.d. of three experiments and triplicate determinations. *$P < 0.01$. **A**, HBECs; **B**, MDA-MB-231 cells

Deng et al (1996) have recently reported that PAI-1 promotes cell detachment by interacting with vitronectin, independent of its activity to function as a protease inhibitor. In our study, although TGF-β1 strongly increased uPA and PAI-1 in MDA-MB-231 cells, it did not inhibit cell proliferation. This is consistent with the work of Keski-oja et al (1988*a* and *b*), which showed that the human lung carcinoma cell line A549 is not growth inhibited by TGF-β1, but responds to TGF-β1 treatment by changes in the expression of matrix components and enhanced proteolytic activity. Additionally, our observations agree with those of Geiser et al (1992) showing that human carcinoma cell lines that are not growth inhibited by TGF-β1 are still capable of responding with increased levels of fibronectin, type IV collagenase and PAI-1. The loss of responsiveness of tumour cells to the antiproliferative effect of TGF-β1 appears to be a frequent event in the progression of malignant tumours (Markowitz and Roberts, 1996). Multiple changes are required for the cells to gain complete resistance to TGF-β1 growth inhibition (Fynan and Reiss, 1993). Recent work has demonstrated that patients suffering from invasive prostatic cancer present a higher plasma TGF-β1 level (Ivanovic et al, 1995), suggesting a role of TGF-β1 in tumour invasion.

In contrast to MDA-MB-231 tumour cells, HBECs were growth inhibited by TGF-β1. Moreover, TGF-β1 decreased uPA activity but augmented PAI-1 antigen level in these cells. TGF-β1 prevented cell detachment of HBECs by inhibiting uPA activity. These results agree with previous studies in fibroblast cells demonstrating that TGF-β1 reduces expression of proteases, such as collagenase and uPA, while increasing expression of protease inhibitors, such as PAI-1 and tissue inhibitor of metalloproteinase (Laiho et al, 1986; Overall et al, 1991). However, Stampfer et al, (1993) have reported that TGF-β1 strongly induces the mRNA and protein synthesis of uPA and PAI-1 in human mammary epithelial cells, but they did not determine the uPA activity.

TGF-β1 is synthesized as a biologically inactive form, which can be converted into the active form by limited proteolysis. Plasmin is one of the factors capable of activating the latent form of TGF-β1. As TGF-β1 is produced by both MDA-MB-231 cells and HBECs (Valverius et al, 1989; Arrick et al, 1990), the enhanced production of plasmin by TGF-β1 would provide a positive feedback loop in the regulation of plasmin-mediated proteolysis in the breast tumour cell line MDA-MB-231, whereas the decreased production of plasmin by TGF-β1 in HBECs would provide a negative feedback in normal breast epithelium.

We have also found that sodium butyrate inhibited both cell proliferation and uPA activity in both HBECs and MDA-MB-231 cells. This is consistent with the finding of Reeder et al (1993), demonstrating that in the human colon carcinoma cell line LIM 2405, sodium butyrate regulates the balance of uPA/PAI-1 in a manner that favours net decreased plasminogen activator activity. Sodium butyrate has been reported to induce cell differentiation of a wide range of tumour cells (Prasad, 1980). It thus may prevent tumour development by inhibiting cell proliferation and invasion as well as by inducing cell differentiation.

In conclusion, our results indicate that (1) while TGF-β1 was a potent inhibitor for cell proliferation and uPA activity in normal cells, it may promote invasion and metastasis of tumour cells by increasing uPA activity and PAI-1 levels; (2) sodium butyrate treatment offers a promising approach to preventing tumour development by inhibiting cell proliferation and invasion. However, recent works have demonstrated that uPA, uPA-R and PAI-1 influence cell adhesion in a process independent of uPA-mediated proteolysis (Waltz and Chapman, 1994; Deng et al, 1996). The possibility of such influence on cell adhesion in our model remains unknown and further studies should be performed to understand the mechanism by which TGF-β1 and other inhibitory agents regulate cell adhesion/migration in the context of the uPA system.

### ACKNOWLEDGEMENTS

This work was supported by Groupement des Enterprises Françaises dans la Lutte Contre le Cancer de Flandres-Artois. We are grateful to Mrs M Stamens for her excellent technical assistance.

### REFERENCES

Achbarou A, Kaiser S, Tremblay G, Ste-Marie LG, Brodt P, Goltzman D and Rabbani SA (1994) Urokinase overproduction results in increased skeletal metastasis by prostate cancer cells *in vivo*. *Cancer Res* **54**: 2372–2377

Andresen PA, Georg B, Lung LR, Riccio A and Stacey SN (1990) Plasminogen activator inhibitors: hormonally regulated serpins. *Mol Cell Endocrinol* **68**: 1–19

Arnoletti JP, Albo D, Granick MS, Solomon MP, Castiglioni A, Rothman VL and Tuszynski GP (1995) Thombospondin and transforming growth factor-beta 1 increase expression of urokinase-type plasminogen activator and plasminogen

© Cancer Research Campaign 1998

activator inhibitor-1 in human MDA-MB-231 breast cancer cells. *Cancer* **76**: 998–1005

Arrick BA, Korc M and Derynck R (1990) Different regulation of expression of three transforming growth factor beta species in human breast cancer cell lines by estradiol. *Cancer Res* **50**: 299–303

Berthon P, Pancino G, De Cremoux P, Roseto A, Gespach C and Calvo F (1992) Characterization of normal breast epithelial cells in primary cultures: differentiation and growth factor receptor studies. *In Vitro Cell Dev Biol* **28**: 716–724

Blasi F (1993) Urokinase and urokinase receptor: a paracrine/autocrine system regulating cell migration and invasiveness. *BioEssays* **15**: 105–111

Bouchet C, Spyratos F, Martin PM, Hacène K, Gentile A and Oglobine J (1994) Prognostic value of urokinase-type plasminogen activator (uPA) and plasminogen activator inhibitors PAI-1 and PAI-2 in breast carcinomas. *Br J Cancer* **69**: 398–405

Delannoy-Courdent A, Fauquette W, Dong-Le Bourhis X, Boilly B, Vandenbunder B and Desbiens X (1996) Expression of c-ets-1 and uPA genes is associated with fibroblast-induced tubulogenesis in normal mammary epithelial cells or with constitutive scattering in cancerous epithelial cells. *Int J Dev Biol* **40**: 1097–1108

Delehedde M, Deudon E, Boilly B and Hondermarck H (1996) Heparan sulfate proteoglycans play a dual role in regulating fibroblast growth factor-2 mitogenic activity in human breast cancer cells. *Exp Cell Res* **229**: 398–406

Deng G, Curriden SA, Wang S, Rosenberg S and Loskutoff D (1996) Is plasminogen activator inhibitor-1 the molecular switch that governs urokinase receptor-mediated cell adhesion and release? *J Cell Biol* **134**: 1563–1571

Desruisseau S, Ghazarossain-Ragni E, Chinot O and Martin PM (1996) Divergent effect of TGFβ on growth and proteolytic modulation of human prostatic cancer cell lines. *Int J Cancer* **66**: 796–801

Ellis V, Behendt N and Danø K (1991) Plasminogen activation by receptor-bound urolinase. A kinetic study with both cell-associated and isolated receptor. *J Biol Chem* **266**: 12752–12758

Fynan TM and Reiss M (1993) Resistance to inhibition of cell growth by transforming growth factor-β and its role in oncogenesis. *Crit Rev Oncogen* **4**: 493–540

Geiser AG, Burmester JA, Webbink R, Roberts AB and Sporn MB (1992) Inhibition of growth by transforming growth factor-β following fusion of two nonresponsive human carcinoma cell lines. *J Biol Chem* **267**: 2588–2593

Gould VE, Koukoulis GK and Virtanen I (1990) Extracellular matrix proteins and their receptors in the normal, hyperplastic and neoplastic breast. *Cell Diff Dev* **32**: 409–416

Ivanovic V, Melman A, David-Joseph B, Valvic M and Geliebter J (1995) Elevated plasma levels of transforming growth factor beta 1 in patients with invasive prostate cancer. *Nature Med* **1**: 282–284

Jankun J, Merrick HW and Goldblatt PJ (1993) Expression and localization of elements of the plasminogen activation system in benign breast disease and breast cancers. *J Cell Biochem* **53**: 135–144

Jones JL, Critchley DR and Walker RA (1992) Alternation of stromal protein and integrin expression in breast: a marker of premalignant change? *J Pathol* **167**: 399–406

Keski-Oja J, Blasi F, Leof EB and Moses HL (1988*a*) Regulation of synthesis and activity of urokinase plasminogen activator in A 549 human lung carcinoma cells by transforming growth factor-β. *J Cell Biol* **106**: 451–459

Keski-Oja J, Raghow R, Sawdey M, Loskutoff DJ, Postlethwaite AE, Kang AH and Moses HL (1988*b*) Regulation of mRNAs for type-1 plasminogen activator inhibitor, fibronectin, and type-I procollagen by transforming growth factor-β. *J Biol Chem* **263**: 3111–3115

Laiho M, Saksela O and Keski-Oja J (1986) Transforming growth factor beta alters plasminogen activator activity in human skin fibroblasts. *Exp Cell Res* **164**: 399–407

Lund LR, Rφmer J, Thomasset N, Solberg H, Pyke C, Bissell M, Danø K and Werb Z (1996) Two distinct phases of apoptosis in mammary gland involution: proteinase-independent and -dependent pathways. *Development* **122**: 181–193

Markowitz SD and Roberts AB (1996) Tumour suppressor activity of the TGF-β pathway in human cancers. *Cytokine Growth Factor* **7**: 93–102

Ossowski L, Russo-Payne H and Wilson E (1991) Inhibition of urokinase-type plasminogen activator by antibodies: the effect on dissemination of a human tumor in the nude mouse. *Cancer Res* **51**: 274–281

Overall CM, Wrana JI and Sodek J (1991) Transcriptional and post-transcriptional regulation of 72-kDa gelatinase, type IV collagenase by transforming growth factor- β1 in human fibroblasts. *J Biol Chem* **266**: 14064–14071

Prasad KN (1980) Butyric acid: a small fatty acid with diverse biological functions. *Life Sci* **27**: 1351–1358

Reeder JA, Dickinson JI, Chevevix-Trench G and Antalis TM (1993) Sodium butyrate differentially modulates plasminogen activator inhibitor type-1, urokinase plasminogen activator, and its receptor in a human colon carcinoma cell. *Teratogenesis, Carcinogenesis and Mutagenesis* **13**: 75–88

Reinartz J, Batrla R, Boukamp P, Fusenig N and Kramer MD (1993) Binding and activation of plasminogen at the surface of human keratinocytes. *Exp Cell Res* **208**: 197–208

Stampfer MR, Yaswen P, Alhadeff M and Hosoda J (1993) TGFβ induction of extracellular matrix associated proteins in normal and transformed human mammary epithelial cells in culture is independent of growth effects. *J Cell Physiol* **155**: 210–221

Valverius EM, Walker-Jones D, Bates SE, Stampfer MR, Clark R, McCormick F, Dickson RB and Lippman ME (1989) Production of and responsiveness to transforming growth factor-beta in normal and oncogene-transformed human mammary epithelial cells. *Cancer Res* **49**: 6269–6274

Vassalli JD and Belin D (1987) Amiloride selectively inhibits the urokinase type plasminogen activator. *FEBS Lett* **214**: 187–191

Waltz DA and Chapman HA (1994) Reversible adhesion to vitronectin linked to urokinase receptor occupancy. *J Biol Chem* **269**: 14746–14750

Yu H and Schultz R (1991) Relationship between secreted urokinase plasminogen activator activity and metastatic potential in murine B 16 cells transfected with human urokinase sense and antisense genes. *Cancer Res* **50**: 7623–7633

Zabrenetzky V, Hariss CC, Steeg PS and Roberts DD (1994) Expression of the extracellular matrix molecule thrombospondin inversely correlates with malignant progression in melanoma, lung and breast carcinoma cell lines. *Int J Cancer* **59**: 191–195

© Cancer Research Campaign 1998